# Nurses, physicians and patients’ knowledge and attitudes about nurse prescribing

**DOI:** 10.1186/s12912-022-00888-0

**Published:** 2022-05-11

**Authors:** Hamidreza Haririan, Deniz Manie Seresht, Hadi Hassankhani, Joanne E. Porter, Lydia Wytenbroek

**Affiliations:** 1 Assistant Professor of Nursing, School of Nursing & Midwifery, Tabriz University of Medical Sciences, Tabriz, Iran; 2 MSc in Nursing, Tabriz University of Medical Sciences, Tabriz, Iran; 3 Professor of Nursing, School of Nursing and Midwifery, Tabriz University of Medical Sciences, Tabriz, Iran; 4 Associate Professor of Nursing, School of Nursing, Midwifery and Healthcare, Federation University Australia, Ballarat, Australia; 5 Assistant Professor of Nursing, University of British Columbia, Vancouver, BC Canada

**Keywords:** Knowledge, Attitude, Nurse Prescribing, Patient, Physician, Critical care

## Abstract

**Background:**

One of the roles that nurses have acquired in recent years is the role of prescribing. This study aimed to investigate the knowledge and attitudes of critical care nurses, physicians and patients about nurse prescribing.

**Methods:**

A descriptive cross-sectional study with the participation of 152 nurses, 53 physicians and 75 patients was carried out. Participants were selected by stratified random sampling from the critical care units of six hospitals in Tabriz, Iran. Demographics and participants’ knowledge and attitudes about nurse prescribing questionnaires were used to collect data. The collected data were analyzed using SPSS-22 software.

**Results:**

The mean scores of total knowledge about nurse prescribing in nurses, patients and physicians’ were 15.41 ± 1.85,16.45 ± 2.31, 14.74 ± 1.7 respectively (from a range of 10 -20), and the mean score of knowledge by physicians was significantly higher than others (*P* = 0.000) and they had more knowledge about nurse prescribing. The mean scores of the attitudes towards nurse prescribing in nurses, physicians and patients were 40.62 ± 3.68, 37.98 ± 5.92 and 39.38 ± 4.39 respectively (from a range of 10 -50). However, the total mean score of attitudes among nurses was significantly higher than others (*P* = 0.000) and nurses had more positive attitudes toward prescribing.

**Conclusion:**

The results showed that the participants have a good understanding and attitudes toward nurse prescribing. Nurse prescribing as a new duty and authority can be considered in providing more effective care by specialist nurses. The results of this study can also be used in the future planning of health policy for nurses to have the right to prescribe and ultimately improve the quality of patient care.

**Supplementary Information:**

The online version contains supplementary material available at 10.1186/s12912-022-00888-0.

## Background

Nurses, constitute the most numerous human resources in the field of health and have many roles and responsibilities [1]. One of the roles that nurses have acquired in recent years with its scope of responsibility increasing day by day is the role of prescribing [2, 3]. There is an increasing number of countries embracing nurses. However, the legal, educational, and organizational conditions in which a nurse prescribes medication vary greatly from country to country. A nurse may only be allowed to prescribe from a limited list of medications under the supervision of a physician to being authorized to prescribe without any restrictions [4].

In the UK in 1990s it became possible for community based nurses to prescribe independently from a limited formulary, thereby facilitating their traditional roles such as wound management. From 2000, further changes in legislation altered the professional restrictions on prescribing, and since May 2006 independent nurse prescribers in England have been able to prescribe any licensed medicine for any medical condition within their competence [5]. Nurse practitioners in the US were entitled to prescribing rights in 1969 and are currently prescribing medications in more than 21 states without physician oversight [6].

In many countries, health policymakers have responded to increasing demand for care due to aging populations and the increasing prevalence of chronic disease, physician shortages, and budget constraints, through strategies such as modernizing roles and combining health professions, including the expansion of the role of nurses [7]. One of the most important role changes has been the endorsement of allied health professionals being able to prescribe [8]. Allied professionals such as pharmacists, and radiologists are joining nurses who can prescribe following appropriate training [9]. Nurses’ ability to prescribe has been a historic development for the profession and an important part of the solution in many health systems in leading countries to improve access and reduce the waiting time for patients to receive medication [10]. Other potential benefits of nurses prescribing are increased continuity of patient care and better access to medication, efficiency in drug delivery and patient comfort, and reduced patient waiting time [10, 11].

In specialist wards, such as the Intensive Care Unit (ICU) and the Cardiac Care Unit (CCU), due to the nature of the ward and the acuity of the patients, nurses have more scientific and practical capabilities which lead to clinically competent nursing staff. Also, due to the critical condition of the patient, it is sometimes necessary for nurses to make quick and sudden decisions to save the patient's life, among which one of these decisions may be prescribing medication or other life-saving care measures which ultimately improve patient outcomes [12].

In a study conducted by Darvishpour et al. (2016) entitled “situational analysis of nurses prescribing context in Iran”, nurses noted incidents whereby prescribing had taken place. Participants who agreed with the implementation of nurses prescribing believed that the community would benefit from the service delivery from nurses and that it would improve health outcomes. Further, nurses were well situated to respond to patient changes quickly and appropriately especially in the absence of physicians [13]. In the study of Babaei et al. (2022) which aimed to determine the attitude and readiness of Iranian nurses towards nurse prescribing, the results showed that nurses had a good preparation and attitude towards nurse prescribing [14].

In Iran, Behvarzes in rural health centers have permission to prescribe in Patient Group Directions (PGD) format. Patient group directions provide a legal structure that allows some health care providers to supply or administer specifed drugs to a group of patients. Behvarzes undergo 2 years of classroom and practical training before beginning work [15]. It should be noted that Iranian critical care nurses sometimes administer medications to critically unwell patients when it has been difficult to gain access to a physician. Nurses assess and examine the patient, administer the drug, and then later consult with the physician to confrm their diagnosis and treatment [16].

Also, there is no legislation in place for nurses to prescribe in Iran and nurse prescribing is already done informally by nurses; However, there is a lack of sufficient and specific information about the attitudes and experiences of critical care’ nurses, physicians, and patients about this phenomenon [17, 18].

This study aimed to investigate the knowledge and attitudes of critical care’ nurses, physicians and patients about nurse prescribing.

## Methods

This cross-sectional descriptive study was conducted with 280 nurses, physicians and patients in critical care units (ICUs and CCUs) in Tabriz, Iran, from July to September 2020. Participants were selected based on a stratified random sampling technique. Accordingly, each of the 6 teaching hospitals of Tabriz was regarded as a strata and its different ICUs/CCUs were taken as classes of that strata. The nurses, physicians and patients in these classes were included in the list of participants based on random sampling. Considering the ratio of the number of nurses, physicians and patients in each hospital to the whole research population, participants were randomly selected as the sample. The inclusion criteria for nurses and physicians included having at least 6 months job experience in ICUs/CCUs and willingness to participate in the study. The exclusion criteria included the reluctance of the nurses or physicians to continue participating in the study. However, the inclusion criteria for patients were adults over 18 years of age, able to complete the questionnaire, and willingness to participate in the study. Patients were excluded if their condition became worse during the study period.

The sample size was determined using the Cochran formula. The total number of nurses and physicians in six teaching hospitals were 548 and 199, respectively. Also, the total number of active beds of these hospitals were 275. By placing these numbers and considering the error level of 5% and *p* = 0.5 in the formula, 152 nurses, 53 physicians, and 75 patients were selected as participants. The response rate for those invited to take part was 100 percent.

The data collection technique was questionnaires and consisted of three parts: demographic information, participants’ knowledge about nurse prescribing and participants’ attitude toward nurse prescribing questionnaires. Demographic information included age, gender, employment status, work experience, academic degree, history of prescribing medication to patients, history of prescribing medication to family members or friends, reasons for prescribing, history of working abroad, physicians and patients’ trust in nurses, patients’ occupation, past hospitalization history, existence of a nurse in the family.

Part two of the questionnaire includes questions that determines the participants’ knowledge about nurse prescribing including 10 items scored based on true (1 point) or false (2 point) responses. The highest score was 20 and the lowest was 10 with a lower score indicating more knowledge about nursing prescribing. The authors of this paper prepared their own questionnaire which was subject to validation prior to its use in the presented study.

Part three of the questionnaire measured the participants’ attitude toward nurses prescribing and included 10 items. The items were scored based on a 5-point Likert scale (1: totally disagree, 2: disagree, 3: no comment, 4: agree, and 5: totally agree). The highest and the lowest scores were 50 and 10, respectively. Higher scores indicated more positive attitudes towards nurses prescribing [19]. The nurse prescribing questionnaire (the initiative survey that improved by the National Independent Evaluation of the Nurses and Midwife Prescribing) including statements related to the variables was used to measure attitude and readiness for nurse prescribing. This questionnaire including 20 items on the two mentioned subscales (10 for attitude, and 10 for readiness) [19]. After obtaining the initial permission from the author, the translation and re-translation process of the questionnaire from English to Persian and Persian to English (by an expert in the English language) was completed; and statements related to attitude selescted for present study.

Content validity of the data collection tool was conducted, the questionnaires was sent to 10 nursing professors, 10 patients, 10 nurses, and 10 physicians who rated the clarity and validity of the content. In addition, Cronbach’s alpha coefficient for the knowledge and attitude subscale was 0.728 and 0.722, respectively. However, Cronbach’s alpha coefficient for the measurement tool was 0.725, which confirmed its reliability.

Ethical approval was obtained from the Ethic Committee of Tabriz University of Medical Sciences (IR.TBZMED.REC.1398.1245). However, methods were performed in accordance with the relevant guidelines and regulations (Declaration of Helsinki).

After the research project was approved by the research ethic committee of Tabriz University of Medical Sciences, the researcher visited the hospitals in different working shifts to brief participants on the research objectives and procedures and then obtained their written informed consent.

The statistical analysis of the data was performed in SPSS V.22. The demographics were analyzed using descriptive statistics. Since the Kolmogorov–Smirnov test showed that the data distribution was normal, ANOVA and the T-test test were employed to compare the mean scores. The significance level for all tests was determined to be *p* < 0.05.

## Results

A total of 280 individuals participated in the study. The sample comprised of critical care nurses (*n* = 152), critical care physicians (*n* = 53), and patients hospitalized in critical care units (*n* = 75). The mean age of participants was 31.40 ± 6.30 for nurses; 35.86 ± 4.80 for physicians; and 38.88 ± 12.01 for patients. More than half of the nurses (77%) had a history of prescribing medication to patients. The majority of the nurses (84.2%) stated that they prescribed medication to patients, and family members based on their knowledge and work experience. The most of the physicians were medical residents (64.2%), and 73.6% of the physicians stated that they have trust in the majority of critical care nurses. However, the most of the patients did not have previous hospitalization history (61.3%), and 54.7% of them stated that they have trust in the majority of critical care nurses. The majority of patients (90.6%) reported receiving prescriptions from their relative nurses (Table 1).

**Table 1:** Participants' characteristics

Characteristics		**Nurses** n (%)	**Physicians** n (%)	**Patients** n (%)
**Gender**	Male Female	59 (38.8) 93 (61.2)	24(45.3) 29 (54.7)	31 (41.3) 44 (58.7)
**Place of work (**ward)	ICU CCU	105 (69.1) 47(30.9)	36 (67.9) 17 (32.1)	65 (86.7) 10 (13.3)
**Work experience year**(mean)		6.7 ± 5.02	3.9 ± 2.40	-
**Age year** (mean)		31.4 ± 6.3	35.8 ± 4.8	38.8 ± 12.0
**Educational level**	Primary Diploma Bachelor Master MD Resident Specialist	- - 142 (93.4) 10 (6.6) - - -	- - - - 4 (7.4) 34 (64.2) 15 (28.4)	9 (12) 12 (6) 25 (33.3) 13 (17.4) - 16 (21.3) -
**Occupation**	Employee Self-employee Housekeeper Retired worker Others	- - - - - -	- - - - - -	19 (25.4) 16 (21.3) 24 (32) 3 (4) 3 (4) 10 (13.3)
**Do you (nurse) prescribe medicine for your patients**?	Yes No	117 (76.9) 35 (23.1)	- -	- -
**Do you (nurse) prescribe medicine for your family**?	Yes No	133 (87.5) 19 (12.5)	- -	- -
**What is the reason if you (nurse) prescribe medicine for your patients and families**?	-I can do prescribing based on my knowledge and work experience -Most of the time the doctor is not available -Doctor will prescribe the same medication I recommend - Boosts my self-confidence and increases patient’s adherence	128 (84.2) 9 (5.9) 11 (7.2) 4 (2.7)	- - - -	- - - -
**History of working abroad** or studying opportunities (**physician**)	Yes No	- -	2 (3.8) 51 (96.2)	- -
**Previous hospitalization** (**patient**)	Yes No	- -	- -	29 (38.7) 46 (61.3)
**Do you (patient) have a nurse among your family members**?	Yes No	- -	- -	26 (34.7) 49 (65.3)
**Has a nurse in your family ever prescribed medicine for you**?	Yes No	- -	- -	68 (90.6) 7 (9.4)
**Did you (patient) feel satisfied with her/his prescription**?	Yes No	- -	- -	49 (65.3) 26 (34.7)
**How much do you trust the work of the nurses** in this ward?	-I trust the majority -I trust some -I do not trust the majority -I do not trust any of them	- - - -	39 (73.6) 14 (26.4) 0 0	41 (54.7) 34 (45.3) 0 0

Most participants did not know that "In countries where nurses are allowed to prescribe, they can prescribe independently" (87% of nurses, 98% of physicians and 88% of patients), but the majority knew that "The nurses prescribing have potential benefits for their patients" (87% of nurses, 98% of physicians and 100% of patients) (Table 2

**Table 2:** Frequency distribution of the participants’ knowledge in the nurse prescribing

Responses	**True**			**False**		
**knowledge**	Nurse n (%)	Physician n (%)	Patient n (%)	Nurse n (%)	Physician n (%)	Patient n (%)
1. Nurses in most developed countries (i.e. UK, United States, Australia, Sweden, New Zealand,.) have the right to prescribe medications	138 (90.8)	33 (62.3)	43 (57.3)	14 (9.2)	20 (37.7)	32 (42.7)
2. The rules for nurse prescribing vary from country to country	142 (93.4)	50 (94.3)	64 (85.3)	10 (6.6)	3 (5.7)	11 (14.7)
3. In the countries where nurses have the right to prescribe, they can prescribe medication from a limited list of drugs	97 (63.8)	29 (54.7)	68 (90.7)	55 (36.2)	24 (45.3)	7 (9.3)
4. In the countries where nurses have the right to prescribe, they can prescribe medication under the supervision of a doctor	117 (77)	30 (56.6)	74 (98.7)	35 (23)	23 (43.4)	1 (1.3)
5. In the countries where nurses have the right to prescribe, they can prescribe independently	20 (13.2)	1 (1.9)	8 (10.7)	132 (86.8)	52 (98.1)	67 (89.3)
6. In the countries where nurses have the right to prescribe, they must have sufficient clinical experience and take a special prescription course	98 (64.5)	33 (62.3)	71 (94.7)	54 (35.5)	20 (37.7)	4 (5.3)
7. The nurses prescribing have potential benefits for patients	132 (86.8)	52 (98)	75 (100)	20 (13.2)	1 (2)	0
8. The nurses prescribing have potential benefits for healthcare systems	110 (72.4)	36 (67.9)	71 (94.7)	42 (27.6)	17 (32.1)	4 (5.3)
9. Nurses in Iran do not have the right to prescribe	141 (92.8)	45 (84.9)	73 (97.3)	11 (7.2)	8 (15.1)	2 (2.7)
10. To meet the urgent needs of patients in critical care units, prescribing by nurses is done informally in Iran	81 (53.3)	3 (5.7)	28 (37.3)	71 (46.7)	50 (94.3)	47 (62.7)

Regarding the attitudes of the participants towards nurses prescribing, the results showed that the most nurses agreed with the statement "prescribing by nurses have positive effects on the nursing profession and discipline" (98%), and "if nurses are allowed to prescribe It could have a positive effect on patient care” (90%). However, the lowest agreement ratio was related to the statement "prescribing by nurses will reduce the patient's referral to various medical professionals "(16.44%). The majority of physicians agreed with the statement "prescribing by nurses will increase patient satisfaction" (94.3%), and "prescribing by nurses could have positive effects on the nursing profession and discipline” (94.3%). And the lowest agreement ratio was related to the statement "prescribing by nurses will reduce the frequency of (chronic) patients visits to the doctor" and "prescribing by nurses will increase patients' access to medicines" (35.9%) (Table 3).

**Table 3:** Frequency distribution of the participants’ attitude in the nurse prescribing

Opinions	Completely agree			Agree			No opinion			Disagree			Completely disagree		
**Attitude**	^a^N n (%)	^a^D n (%)	^a^P n (%)	N n (%)	D n (%)	P n (%)	N n (%)	D n (%)	P n (%)	N n (%)	D n (%)	P n (%)	N n (%)	D n (%)	P n (%)
1. Nurses should be allowed to prescribe medications	53 (34.9)	32 (60.4)	15 (20)	63 (41.4)	4 (7.5)	57 (76)	36 (23.7)	15 (28.3)	1 (1.3)	0	2 (3.8)	2 (2.7)	0	0	0
2. Nurse prescribing would have a positive impact on patient care	77 (50.7)	0	16 (21.3)	60 (39.5)	28 (52.8)	58 (77.3)	15 (9.9)	19 (35.8)	0	0	4 (7.5)	1 (1.3)	0	2 (3.8)	0
3. Nurse prescribing would result in financial savings	40 (26.3)	18 (34)	33 (44)	81 (53.3)	16 (30.2)	13 (17.3)	31 (20.4)	18 (34)	29 (38.7)	0	1 (1.9)	0	0	0	0
4. Nurse prescribing would enhance patient compliance	42 (27.6)	18 (34)	25 (33.3)	81 (53.3)	16 (30.2)	11 (14.7)	27 (17.8)	13 (24.5)	35 (46.7)	2 (1.3)	4 (7.5)	4 (5.3)	0	2 (3.8)	0
5. There is a need for nurse prescribers who provide care for patients with chronic disease (diabetes,..)	40 (26.3)	0	41 (54.7)	93 (61.2)	15 (28.3)	32 (42.7)	19 (12.5)	22 (41.5)	1 (1.3)	0	16 (30.2)	1 (1.3)	0	0	0
6. The introduction of the nurse prescribing initiative would reduce the number of health care professionals a patient must interact with	17 (11.2)	0	4 (5.3)	65 (42.8)	1 (1.9)	48 (64)	45 (29.6)	49 (92.5)	2 (2.7)	25 (16.4)	2 (3.8)	19 (25.3)	0	1 (1.9)	2 (2.7)
7. The introduction of nurse prescribing would enable patients to access medication quicker	55 (36.2)	19 (35.8)	7 (9.3)	80 (52.6)	15 (28.3)	48 (64)	16 (10.5)	0	20 (26.7)	1 (0.7)	18 (34)	0	0	1 (1.9)	0
8. The introduction of the nurse prescribing initiative would increase patient satisfaction levels	54 (35.5)	50 (94.3)	5 (6.7)	66 (43.4)	0	50 (66.7)	32 (21.1)	2 (3.8)	18 (24)	0	0	2 (2.7)	0	1 (1.9)	0
9. Nurse prescribing would reduce the need for patients with long-term illnesses to return to see their doctor as frequently as previously	11 (7.2)	19 (35.8)	4 (5.3)	75 (49.3)	15 (28.3)	33 (44)	51 (33.6)	0	21 (28)	15 (9.9)	18 (34)	14 (18.7)	0	1 (1.9)	3 (4)
10. The introduction of nurse prescribing would have positive benefits for the nursing profession	68 (44.7)	50 (94.3)	32 (42.7)	81 (53.3)	0	0	2 (1.3)	2 (3.8)	0	1 (0.7)	0	43 (57.3)	0	1 (1.9)	0

^a^ *N* nurses, *D* physicians, *P* patients

In addition, the majority of patients agreed with the statement "if nurses are allowed to prescribe that could have a positive effect on patient care" (98.6%), and "for the care of chronic patients (diabetic, dialysis, rheumatic, cardiac …) we need nurses who have the authority to prescribe” (97.4%). The lowest agreement ratio was related to the statement “prescribing by nurses will reduce the patient's referral to various medical professionals "(57.3%) (Table 3).

In addition, the results showed that the mean score of total knowledge about nursing prescribing by physicians is higher than nurses and patients, and this difference is statistically significant (*P* = 0.000). Also, the mean score of the total attitude towards nurse prescribing among nurses is higher than physicians and patients, and this difference is also statistically significant (*P* = 0.000) (Table 4).

**Table 4:** Participants’ knowledge and attiudes about nurse prescribing

Variable	Participants	Min	Max	Mean ± SD	P -Value
**Knowledge**	**Nurses**	10	18	15.41 ± 1.85	df = 2
	**Physicians**	10	17	14.74 ± 1.70	f = 7.92
	**Patients**	12	19	16.45 ± 2.31	*p* = 0.000
	Total	12	21	15.43 ± 1.98	
**Attitudes**	**Nurses**	35	50	40.62 ± 3.68	df = 2
	**Physicians**	21	44	37.98 ± 5.92	f = 12.36
	**Patients**	31	50	39.38 ± 4.05	*p* = 0.000
	Total	21	50	39.79 ± 4.39	

## Discussion

The aim of this study was to investigate the knowledge and attitudes of 280 critical care nurses, physicians and patients towards nurse prescribing in teaching hospitals in Tabriz, Iran.

The results showed that nurses have a relatively high level of knowledge about nurse prescribing and they have a positive attitude towards this issue. Most of the nurses who participated in this study believed that prescribing by nurses could have positive effects on patient care and the nursing profession. Consistent with these results, Haririan et al. (2021) also showed that nursing students had a positive attitude toward nurses prescribing. However, almost half of the participants stated that they did not have a good knowledge of pharmacology [18]. In Stenner and Courtenay study (2008), nurses were well aware of nurse prescribing and reported several benefits, including faster access to treatment, improved quality of care, appropriate medication prescription, improved safety, improved nurse-patient relationship, and increased efficiency [20]. Zarzeka et al. (2017) in a cross-sectional study entitled "Nurse prescribing. Knowledge and attitudes of polish nurses in the eve of extending their professional competences “mentioned that nurses have a positive attitude towards nurse prescribing and believed that introducing the role of nurse prescribing increased the nurse's control over the treatment process [21].

The results of the study showed that critical care’ physicians had a relatively high knowledge and positive attitude towards nurses prescribing. More than half of the physicians participating in the study believed that nurses in critical care units should be allowed to prescribe, and the majority said that nurse prescribing increased the patient satisfaction and could have positive effects on the nursing profession. In a qualitative study conducted by Shannon and Spence (2011) with the aim of determining the attitudes and views of general practitioners and specialist physicians about the role of nurses prescribing for heart failure, data were collected in one primary health-care centre and one teaching hospital in the west of Scotland, UK (where there is legislation in place and training to support nurse prescribing), and findings showed that physicians were highly aware of nurses prescripting medications and supported the practice. Both groups of physicians agreed on its benefits for patient care and increasing patient satisfaction [22].

Zarzeka et al. (2019) in a study entitled "Nurse prescribing: Attitudes of medical doctors towards expanding professional competencies of nurses and midwives “ showed that most physicians believe that nurses and midwives do not yet have sufficient experiences to prescribe medications, and nurses and midwives can only prescribe medications that have already been prescribed by a doctor [23]. This is not consistent with the findings of the current study.

However, the results showed that the patients hospitalized in critical care units have a moderate to high knowledge and attitude towards nurses prescribing. The majority of them stated that nurses prescribing could have positive effects on patient care. The study of Banicek (2013) which investigated the attitudes of postoperative patients towards nurse prescribing in London (where staff and patients have experienced nurse prescribing and there is legislation in place and training to support nurse prescribing), showed that patients had a positive attitude and high confidence in nurse prescribing [24]. In another study conducted in 2008 entitled”patients’ attitudes toward, and information needs in relation to, nurse prescribing in rheumatology”, showed that the participating patients had a relatively high confidence in the prescription abilities of nurses, and stated that they would most likely use those drugs [25]. In another study, patients believed that nurses prescriptions provided better and more efficient access to skin care services [26].

In this study, the mean score of the total knowledge of physicians was higher than nurses and patients and the mean score of the total attitudes among nurses were higher than physicians and patients, and these differences were statistically significant. In a qualitative study (Jones et al. 2007) that aimed to examine the experiences of patients, mental health nurses and psychiatrists regarding nurse prescribing, the results showed that participants from all three groups had a positive attitude toward nurse prescribing [27]. Badnapurkar et al. (2018) in a study aimed to examine the attitudes of nurses and psychiatrists about the role of nurse prescribing, showed that in the five subscales (general beliefs, impact, use, training and supervision), both nurses and physicians had a relatively positive attitude towards nurse prescribing. Also, compared to psychiatrists, nurses were more confident in the wide range of clinical situations in which a nurse prescription could be used (such as acute hospitalization and substance use) [28].

## Conclusion

The results of the present study showed that the knowledge and attitude of critical care nurses, physicians and patients around nurse prescribing are in general positive, and all of them had a good knowledge and a positive attitude towards this advance practice for nursing professionals. The recent master nursing curriculum alterations in Iran (the number of nursing masters has reached to 10, and all students in this specialized level are required to pass 1–2 credit hours pharmacology in their course), which indicates the specialization of nursing and the further development of the role to include nurse prescribing is considered a set in the right direction to enhance the nursing profession.

Nurse prescribing as a new duty and authority can be considered in providing more effective care by specialist nurses. The results of this study can also be used in the future planning of health policy for nurses to have the right to prescribe and ultimately improve the quality of patient care.

## Supplementary Information

**Additional file 1: **Data availability.

## Data Availability

All data analysed during this study are included in this published article [Supplementary File: Data availability.xls].
